# Clinical outcomes of a two-piece zirconia dental implant system

**DOI:** 10.1186/s40729-026-00717-y

**Published:** 2026-09-18

**Authors:** Thomas Mehnert, Michael Noack, Philip Simon, Florian Beuer, Stefano Pieralli

**Affiliations:** 1 Private Practice for Oral and Maxillofacial Surgery, Neumarkt 36-38, 50667 Cologne, Germany; 2 Center for Dental, Oral and Maxillofacial Medicine, Medical Faculty, University of Cologne, Kerpener Str. 62, 50937 Cologne, Germany; 3 Department of Prosthodontics, Geriatric Dentistry and Craniomandibular Disorders, Charité - Universitätsmedizin Berlin, Corporate Member of Freie Universität Berlin and Humboldt-Universität zu Berlin, Aßmannshauser Str. 4-6, 14197 Berlin, Germany

**Keywords:** Alveolar bone loss, Clinical study, Complications, Oral implants, Survival rate, Zirconium dioxide

## Abstract

**Background:**

Data on market-available two-piece zirconia (ZrO₂) dental implants are limited. Therefore, the aim of this retrospective study was to evaluate the clinical performance of a commercially available ZrO₂ implant system over a 24-month period.

**Materials and methods:**

In a single private dental office, 35 patients received 63 two-piece ZrO₂ implants for the support of single crowns (SC), fixed dental prostheses (FDP), and overdentures between September 2017 and October 2021. To ensure cohort homogeneity, 31 patients, which received 44 implants to support SCs, were selected for statistical analysis. An anonymized database was used to examine survival and complication rates, marginal bone loss (MBL), and soft tissue parameters. Patients were followed within a defined recall period of at least two years. Descriptive statistics (95% CI) were calculated for MBL, with paired t-tests and one-way ANOVA for comparisons. Associations and clinical parameters were analyzed using Chi-square/Fisher’s exact, paired t-test, and Wilcoxon tests (*p* ≤ 0.05).

**Results:**

During the observation period, no implant losses or implant fractures occurred. The mean MBL was 0.24 ± 0.58 mm at 6 months, 0.18 ± 0.62 mm at 12 months, and 0.20 ± 0.66 mm at 24 months. These values were statistically within the range of random variation. MBL differed between the maxilla and mandible, but not significantly. Probing depths increased significantly over time but did not exceed 2.78 ± 0.26 mm. Bleeding on probing mean values showed a continuous decrease from 9.1 ± 13.7% at baseline to 1.1 ± 4.3% after 24 months, with significant differences between baseline and 24 months, as well from 6 to 24 months (*p* = 0.002 and *p* = 0.003 respectively).

**Conclusion:**

Over 24 months, two-piece ZrO₂ implants exhibited a 100% survival rate with no observed fractures. MBL was reduced, especially in augmented sites. The presented data support the short-term suitability of this implant system for single-unit restorations.

**Clinical trial number:**

Not applicable.

## Introduction

Two-piece titanium implants represent the current standard in implant dentistry and are supported by numerous prospective long-term studies [2, 13, 23]. In recent years, zirconium dioxide (ZrO₂) implants have gained increasing clinical attention and, according to a Delphi study by the European Association for Osseointegration, are expected to occupy a share of the dental implant market alongside titanium implants [51].

ZrO₂ implant systems are primarily composed of either yttria-stabilized tetragonal zirconia polycrystal (Y-TZP) or alumina-toughened zirconia (ATZ) [50]. Due to their tooth-like white color, ZrO₂ implants may offer esthetic advantages, particularly in situations with thin peri-implant soft tissues [1, 4, 25]. Their remarkable biocompatibility as well as low plaque affinity, and reduced inflammatory response of the peri-implant soft tissues are further advantages compared to titanium implants [16, 52, 55]. Kajiwara et al. observed significantly higher gingival blood flow around ZrO₂ compared to metal abutments, potentially supporting their immune function [27]. Finally, by providing a barrier at the implant–soft tissue interface, ZrO₂ implants may help reduce the risk of peri-implantitis [30, 35, 54]. Current clinical data are however, still too limited to allow a clear understanding of peri-implantitis with ZrO₂ implants [56].

Biotribocorrosion is a mechano-chemical process, that leads to the release of titanium ions and particles [19, 41]. Fully oxidized and sintered ZrO₂ exhibits high corrosion resistance, which may be advantageous especially in medically compromised patients [42]. Animal studies have demonstrated comparable osseointegration between ZrO₂ and titanium implants in terms of bone to implant contact, removal torque and push-out analyses [20, 21, 40, 48]. From a mechanical standpoint, the fracture resistance of ZrO₂ implants is influenced by design and material, with 1-piece and ATZ implants showing superior performance compared to two-piece and Y-TZP, whereas abutment preparation reduces strength [6].

Since the first clinical reports on ZrO₂ implants [8, 37], research on their use has increased substantially over the past two decades. However, most information involve one-piece implant systems, while clinical outcomes for market-available two-piece implant systems remain scarce [56]. Compared with monobloc designs, two-piece systems offer greater prosthetic versatility, retrievability during maintenance and the possibility of a submerged healing phase.

The objective of this study was to evaluate the implant survival rate, marginal bone loss (MBL), and additional clinical parameters—such as probing depth (PD) and bleeding on probing (BOP)—of a commercially available, two-piece, screw-retained ATZ implant system over a two-year period.

## Materials and methods

### Design of the study

This study was conducted as a single-center retrospective investigation. Approval to conduct this investigation was granted by the Ethics Committee of the Medical Association of Nordrhein, Germany (processing number t2L/2023). Consent to participate declaration was provided by all participants. Thirty-five patients with a total of 63 implants met the predefined inclusion criteria. The prosthetic restorations were distributed as follows: 44 implants were restored with single crowns (SC), 13 served as abutments for fixed partial dentures (FDP), and six were used to support overdentures. Due to intergroup heterogeneity and the limited sample sizes of the two smaller cohorts, statistical analyses were restricted to implants restored with SCs to ensure robust and reliable evaluation. This study was conducted and reported in accordance with the STROBE statement for observational cohort studies [57].

### Investigated implant system, surgical and prosthetic procedures, follow-ups

Two-piece ZrO₂ implants (Zeramex XT, CeramTec Schweiz GmbH, Spreitenbach, Switzerland) were evaluated in this study. Zeramex XT implants are made from ATZ, and are created by subtractive manufacturing. Following the final shaping, no additional thermal processes, such as sintering or post-processing, are applied. The endosseous portion is cylindric-tapered and features a rough microstructure (Zerafil, CeramTec Schweiz GmbH) produced by sandblasting with aluminum oxide and etching with hypophosphorous acid. The implant collar region has a total height of 1.6 mm, comprising a 1.0 mm acid-etched portion and a 0.6 mm machined, smooth surface. A distinctive feature of the system is the non-metallic, retrievable VICARBO^®^ screw, made from carbon fiber-reinforced high-performance polymer.

The surgical protocol strictly followed the manufacturer’s guidelines. In two cases, immediate implant placement was performed, and autologous bone was used to fill the peri-implant gap. In all other cases, delayed implant placement was adapted, from 6 months after tooth extraction. In the mandible, careful full-thickness flap elevation was carried out without vertical incisions. In the maxilla, vertical incisions were occasionally required to improve intraoperative visibility and flap mobility. Implant insertion was performed manually with an insertion torque of up to 35 Ncm. Lateral bone augmentation was performed as a single-stage procedure with simultaneous implant placement, in cases of exposed implant threads or < 2 mm vestibular bone lamella. For this purpose, a particulate bone graft mixture was applied under gentle compression. The bone mixture consisted of approximately 60% autologous and 40% xenogeneic bone (Bio-Oss^®^, Geistlich Pharma AG, Wolhusen, Switzerland) in combination with autologous blood. A membrane was consistently applied to protect the graft (Jason^®^ membrane, botiss biomaterials GmbH, Zossen, Germany; Distributed by Institut Straumann AG, Basel, Switzerland). Autologous bone chips were harvested from the mandibular retromolar area using a sterile single-use Safescraper device (Meta Technologies S.r.l., Reggio Emilia, Italy; distributed by Geistlich Pharma AG). In cases presenting a risk of membrane displacement, fixation was achieved using two Frios membrane tacks (Dentsply Sirona Deutschland GmbH, Bensheim, Germany).

When in presence of advanced vertical atrophy of the maxillary alveolar ridge, an external sinus floor elevation was performed. In cases with a vertical residual bone height of less than 4 mm in the posterior maxilla, a two-stage approach was chosen. In these cases, exclusively autologous bone harvested from the facial bony wall was used. The lateral window was covered with the aforementioned membrane without additional fixation. After implant placement, atraumatic wound closure was performed. In all surgical procedures, non-resorbable sutures (Resorba Medical GmbH, Nuremberg, Germany) were used, and were removed postoperatively after 8–10 days. Follow-up examinations were conducted every four weeks until prosthetic delivery.

In the two cases of immediate implant placement, only adaptation sutures were placed for esthetic reasons, and the implants healed without functional loading. In all other cases, the implants underwent submerged healing. Furthermore, provisional restorations were provided without involving the inserted implants, either as tooth-supported restorations using the adjacent dentition or as removable, mucosa-supported prostheses. At the time of implant uncovering, conventional healing abutments were placed in all cases. Implant uncovering was planned after 4 months, and after 6 months in cases involving sinus lift procedures. To transfer the implant position to the lab, conventional open-tray impressions were taken two weeks after implant uncovering, followed by the fabrication of a master model. Definitive restorations were fabricated as monolithic ZrO₂ SCs and FDPs (IPS e.max ZirCAD, Ivoclar, Schaan, Liechtenstein) and cemented onto ATZ abutments in the laboratory using Panavia 21 (Kuraray Noritake, Hattersheim, Germany). Thereafter, the restorations were secured with the VICARBO^®^ screw at a torque of 25 Ncm. The screw access channel was subsequently sealed with composite. All overdentures were designed as telescopic prostheses. The primary components were individually fabricated from ATZ, while polyetheretherketone (PEEK) was used for the secondary components. Treatment, follow-up examinations, and data analyses were conducted by a single practitioner (T.M.).

### Eligibility criteria

**Fig. 1:**
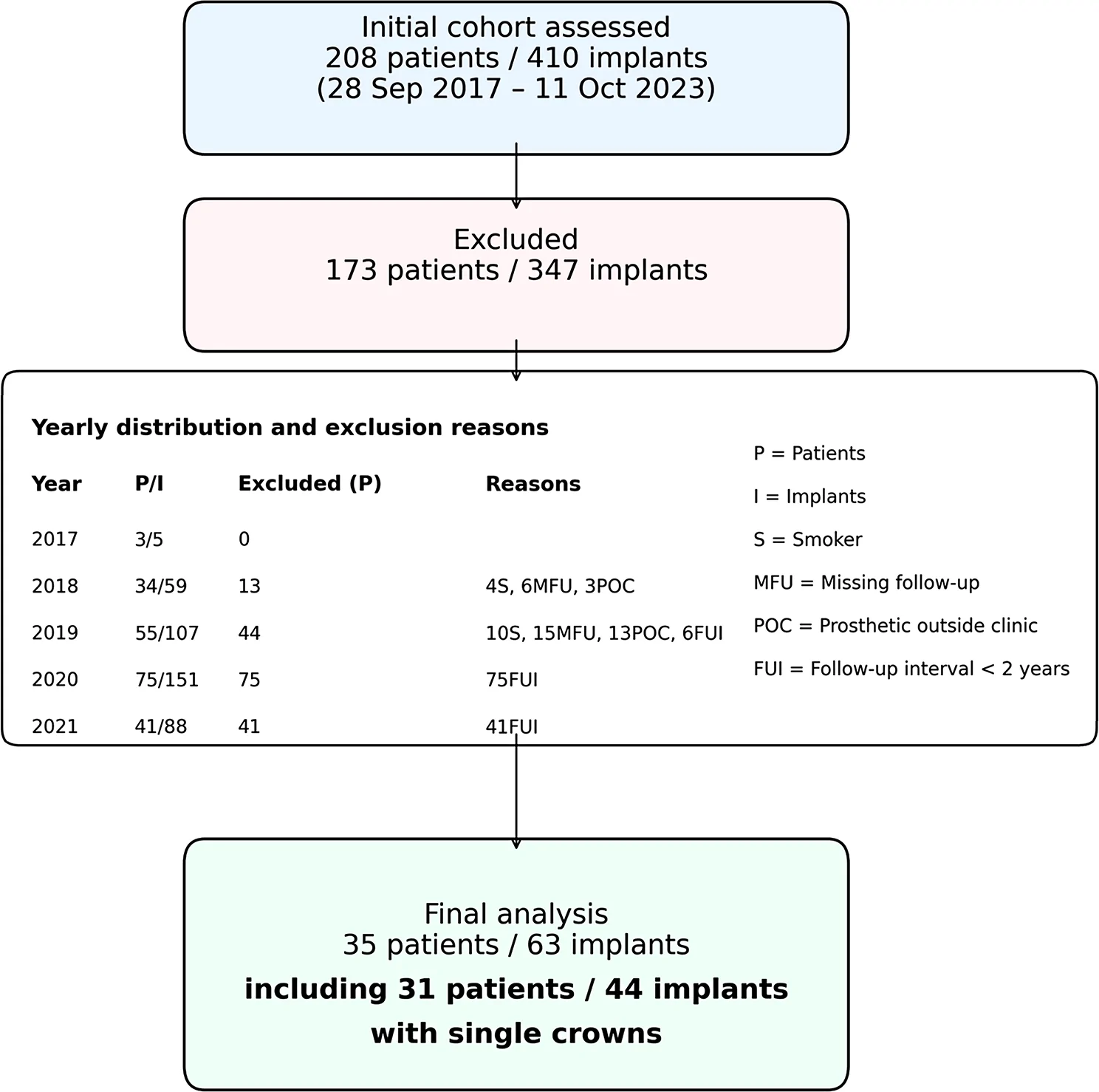
Schematic illustration of the selection process of the study cohort

Patient selection included adults aged ≥ 18 years, irrespective of gender. After providing written informed consent to participate in the study, patient data were collected, anonymized, and recorded in a centralized database for analysis (Fig. 1). Participants had to meet the following inclusion criteria:

- Non-smokers (smoking status was determined by self-report at the initial consultation and recorded in the anonymised database)
- Implant exposure occurred at least 2 years prior
- Attendance at all three recall appointments (6, 12, and 24 months after implant placement)
- Implants are prosthetically restored

### Radiographic evaluation

**Fig. 2:**
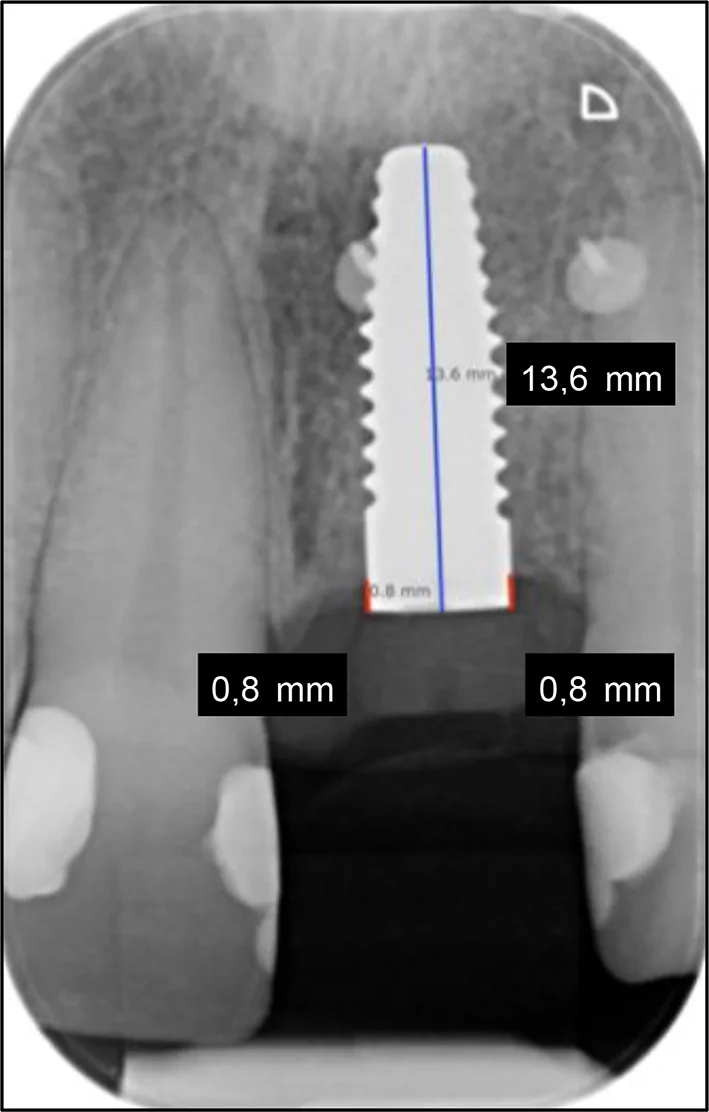
Illustration of the MBL measurement procedure (blue line = implant length; red lines = first bone contact from the implant shoulder, mesially and distally). Two membrane tacks following lateral bone augmentation are visible

Standardized periapical radiographs were obtained using the right-angle technique with the x-ray unit (Sirona Heliodent DS, Dentsply Sirona), employing VistaScan storage phosphor plates 2+/0+ (Dürr Dental, Bietigheim-Bissingen, Germany) and the VistaScan Mini View 2.0 phosphor plate scanner (Dürr Dental). The fixation of the storage plates intraorally was performed using the XCP-DS sensor holder (Dentsply Sirona). Radiographic evaluations were conducted on the day of implant exposure (radiological baseline) as well as at 6, 12, and 24 months after prosthetic delivery. Radiological evaluation was performed using DBSWIN Imaging Software (Dürr Dental). MBL was determined as the difference between the measurements at the defined time points and the marginal bone contact at baseline (Fig. 2). Bone resorption was indicated by positive values, whereas bone apposition was represented by negative values. All measurements were performed by a single calibrated examiner (T.M.). To assess intra-rater reliability, 30 radiographs were randomly selected and re-evaluated by the same examiner four weeks after the initial assessment. To determine inter-rater reliability, the same 30 radiographs were independently assessed by a second calibrated examiner (P.S.). The intraclass correlation coefficient (ICC) was calculated using a two-way mixed-effects model for absolute agreement (single measures). Both intra- and inter-rater reliability demonstrated excellent agreement (ICC = 0.97 and 0.95, respectively).

### Clinical examinations

Clinical parameters of the peri-implant mucosa included PD and BOP. The classification of healthy peri-implant mucosa, peri-implant mucositis, and peri-implantitis was based on the criteria established by the World Workshop on Periodontology by the American Academy of Periodontology (AAP) and the European Federation of Periodontology (EFP). Peri-implant mucositis was defined by positive BOP at more than one measurement site on the implant, erythema, swelling, and/or suppuration. Peri-implantitis was defined by positive BOP at more than one site, probing depths > 6 mm, and bone loss > 2 mm [5, 28, 43, 53]. Implant success after two years was defined as: PD ≤ 4 mm, absence of BOP, no suppuration, and a maximum marginal bone loss of 1.7 mm [5]. Both biological and technical complications were recorded.

### Statistical analysis

Descriptive statistics were initially performed based on the anonymized database. MBL and gain were analyzed with 95% confidence intervals (CI) for all cases, separately for mesial and distal measurements, as well as mean values (MW) considering sample size (N) and standard deviation (SD). The paired Student’s t-test was subsequently applied. For the analysis of mean values in patients with additional bone augmentation over time, a one-way ANOVA was conducted using ONEWAY descriptive statistics. Qualitative analysis of bone loss/gain relative to sex and implant location (maxilla/mandible) was performed using contingency tables with the Chi-square test or Fisher’s exact test where appropriate. The significance level was set at 95% (*p* = 0.05). Probing depth values were analyzed with the paired t-test, and BOP values were assessed using the nonparametric Wilcoxon test. All statistical analyses were conducted using SPSS Statistics version 29.0 (SPSS Inc., Chicago, USA).

## Results

**Fig. 3:**
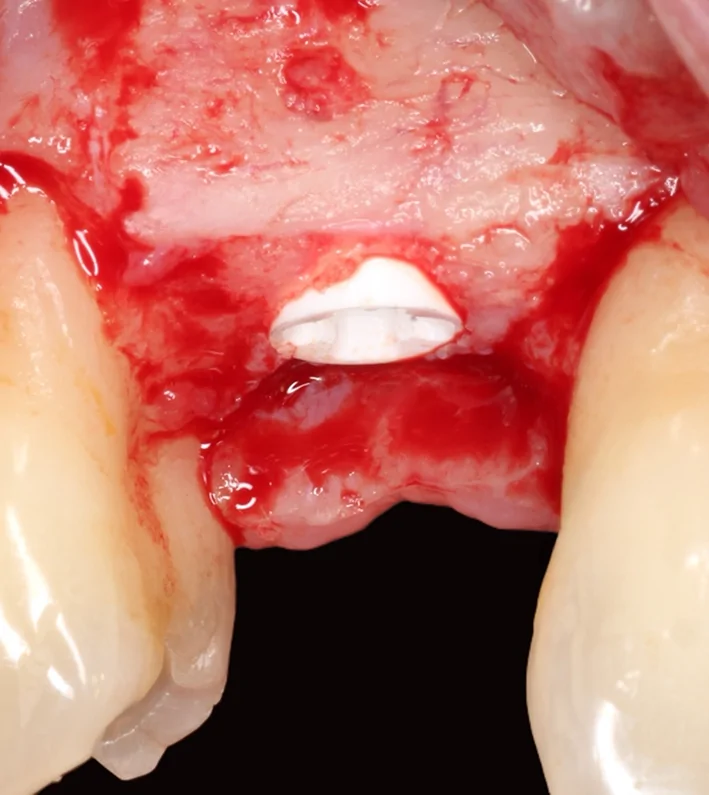
Full-thickness mucoperiosteal flap elevated prior to installation of the Zeramex XT implant (diameter 4.2 mm, length 10 mm) in the posterior mandible. Careful flap management without vertical incisions was employed to preserve periosteal blood supply

**Fig. 4:**
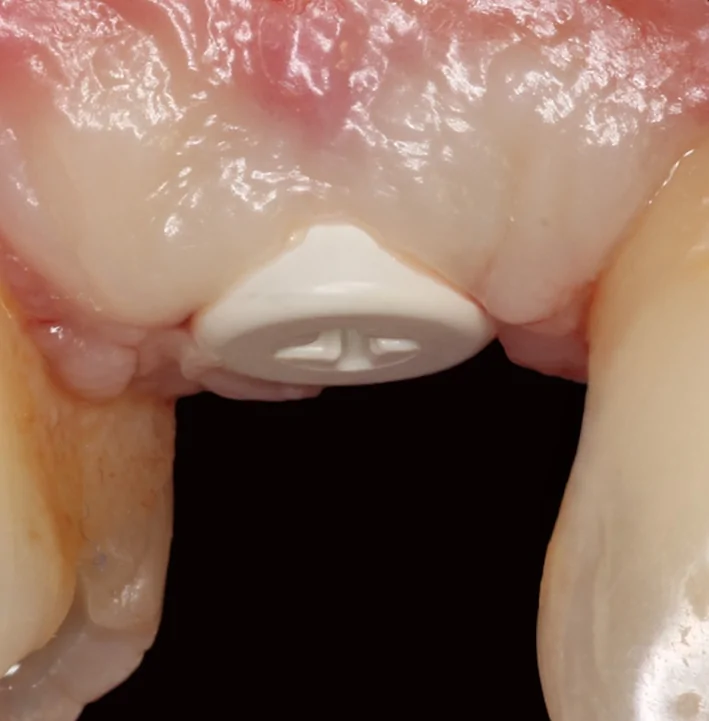
A 4 mm healing abutment placed at the time of implant exposure (second-stage surgery) to initiate soft tissue conditioning prior to impression-taking. The implant had been submerged for approximately 4 months following placement

**Fig. 5:**
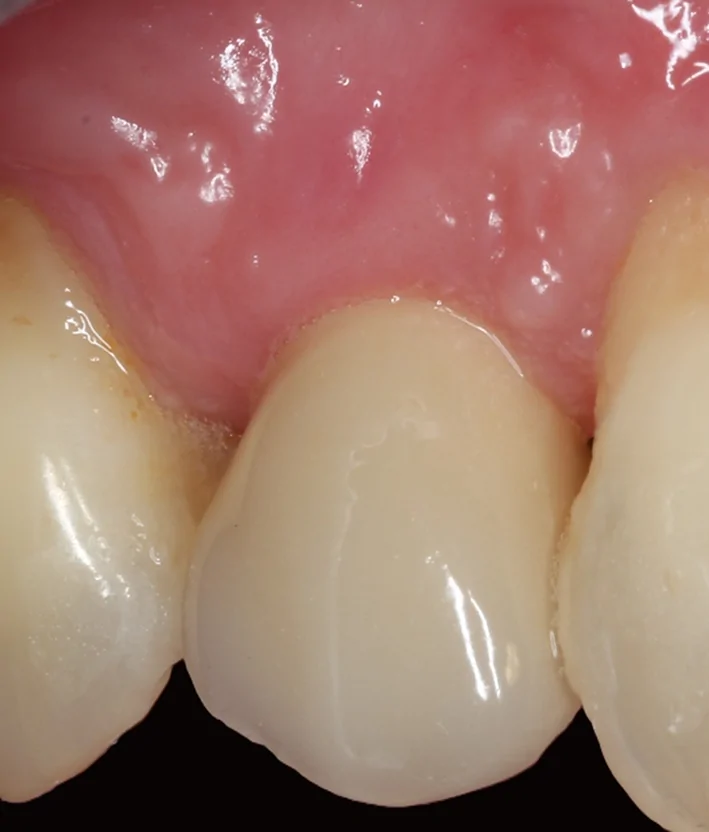
Clinical view six months after prosthetic delivery of a CAD/CAM monolithic ZrO_2_ crown (IPS e.max ZirCAD, Ivoclar) in the posterior region. Peri-implant soft tissue contour and colour are clinically unremarkable

Of the 31 patients receiving SCs, 21 were female (67.7%) and 10 were male (32.3%). Male patients were, on average, older (59.6 years; range: 40.7–72.7 years) than female patients (55.7 years; range: 32.5–80.8 years). The age differences were not statistically significant. The mean age of all patients at the time of implantation was 59.3 years (range: 32.5–80.8 years). A total of 20 implants (45.5%) were placed in the maxilla and 24 implants (54.5%) in the mandible (Figs. 3, 4 and 5). The distribution by tooth groups is shown in Table 1, and implant characteristics are detailed in Table 2. Twenty-two patients received one implant and eleven patients two implants.

**Table 1:** Distribution of 44 implants by intraoral localization

Tooth Group	Frequency	Percentage (%)
Anterior	7	15.9
Premolar	12	27.3
Molar	25	56.8
**Total**	**44**	**100.0**

**Table 2:** Distribution of implant sizes used (*n* = 44)

Implant Characteristics (mm)	Number (*n*)	Percentage (%)
Diameter 4.2	29	65.9
Diameter 5.5	15	34.1
**Total**	**44**	**100**
Length 10	27	61.4
Length 12	17	38.6
**Total**	**44**	**100**

Additional bone augmentation during implant installation was carried out at 22 implants (50%). The distribution of augmentation procedures across the 44 implants is presented in Table 3.

**Table 3:** Frequency of bone augmentation procedures

Surgical procedure	Number (*n*)	Percentage (%)
No augmentation	22	50.0
Filling of the peri-implant gap (immediate implants)	2	4.5
Lateral augmentation	15	34.1
Sinus lift	5	11.4
**Total**	**44**	**100**

### Radiographic evaluation

**Fig. 6:**
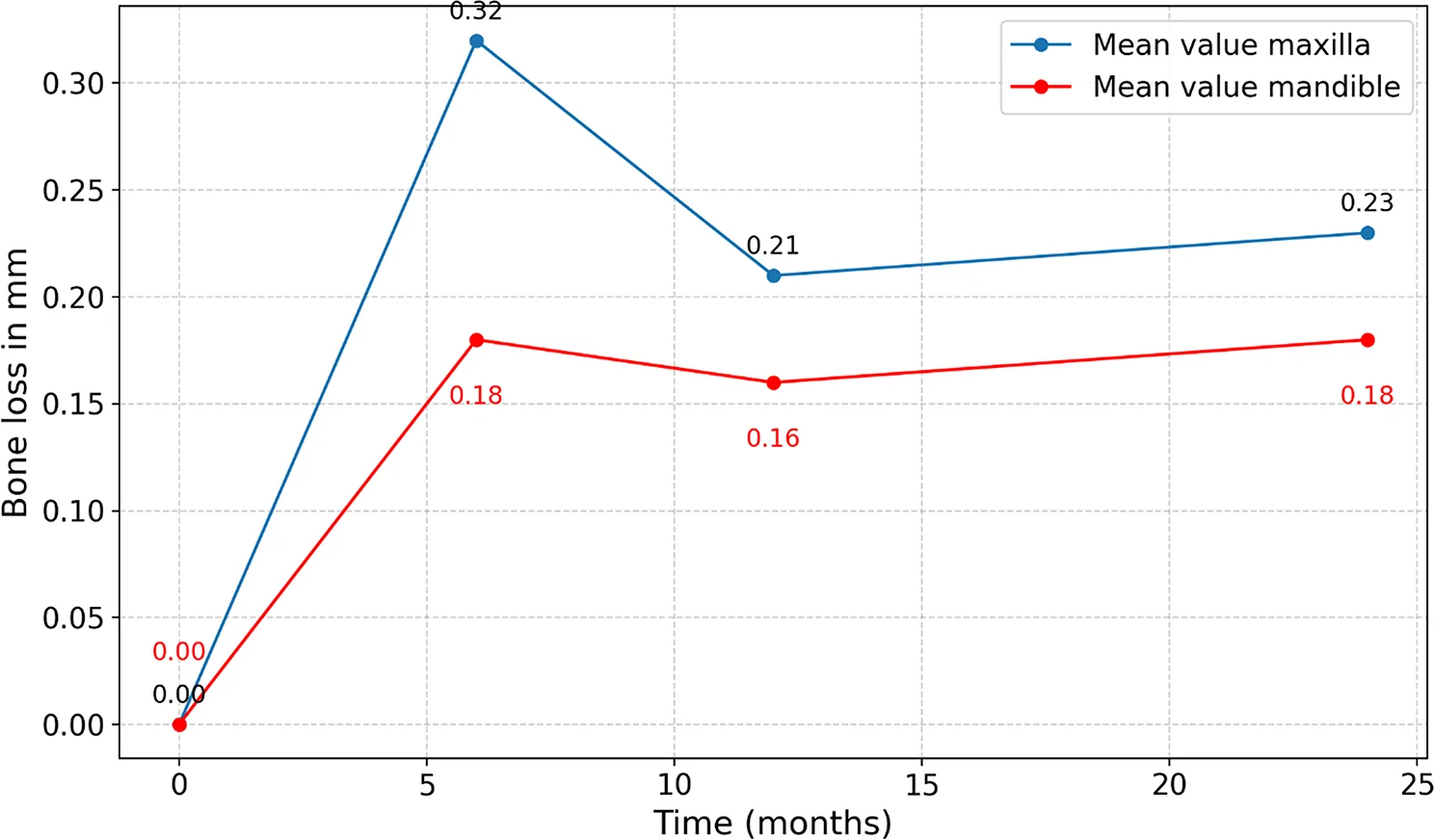
MBL in the maxilla and mandible at the specified time intervals (6, 12, and 24 months) (95% CI)

**Fig. 7:**
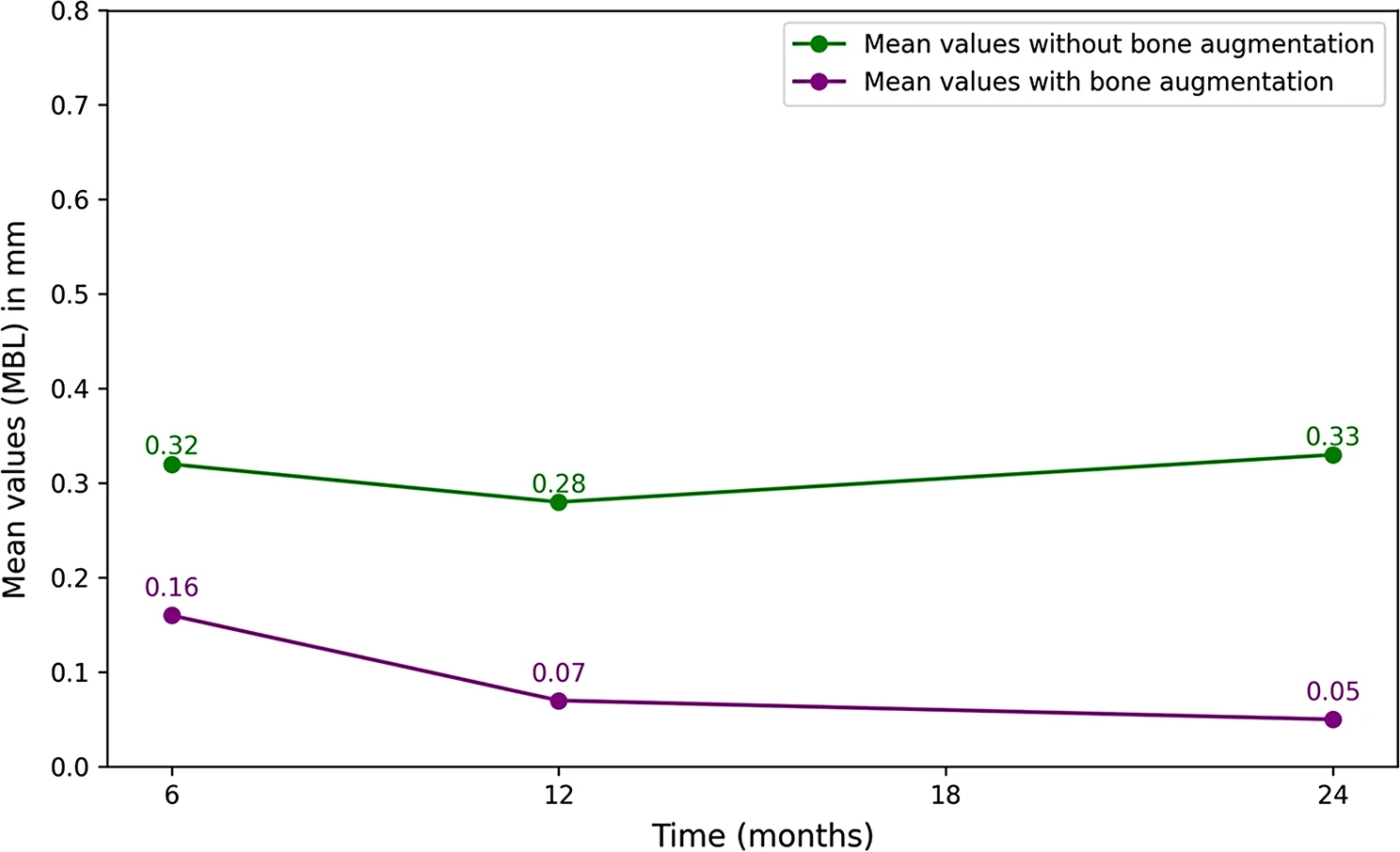
MBL in implants with and without additional bone augmentation (95% CI)

The mean MBL values were 0.24 ± 0.58 mm at 6 months, 0.18 ± 0.62 mm at 12 months, and 0.20 ± 0.66 mm at 24 months. When considering MBL across the different time points, no statistically significant differences were detected by t-test (*p* ≥ 0.062). Figure 6 illustrates the MBL values divided by maxilla and mandible over the 6, 12-, and 24-month periods. In the maxilla, the mean values were 0.32 mm, 0.21 mm, and 0.23 mm, respectively, while in the mandible they were 0.18 mm, 0.16 mm, and 0.18 mm. Statistical analysis using contingency tables combined with the Chi-square test and Fisher’s exact test revealed that the differences in bone loss between the maxilla and mandible at all examined time points were not statistically significant and thus attributable to random variation. No statistically significant association was found between sex and jaw location. In other words, the distribution of implants in the maxilla and mandible between female and male patients showed no differences according to qualitative analysis using the Chi-square test and Fisher’s exact test. The analysis of mean bone loss for patient groups with and without additional bone augmentation is presented in Fig. 7.

The analysis of mean values for the 22 cases with additional bone augmentation at 6, 12, and 24 months using a one-way ANOVA revealed that differences between groups with and without bone augmentation fall within the range of random variation. However, it was observed that cases with bone augmentation generally exhibited less marginal bone loss. These differences were not statistically significant (Table 4).

**Table 4:** Association between bone augmentation and bone loss at different follow-up intervals (*n* = 44)

Variable	Outcome	Group 1: Implant placement with bone augmentation (n=22)	Group 2: Implant placement without bone augmentation (n=22)	p-value
Bone loss – 6 months	Yes	11 (50.0%)	17 (79.2%)	0.059
	No	11 (50.0%)	5 (20.8%)	
Bone loss – 12 months	Yes	12 (55.0%)	16 (70.8%)	0.352
	No	10 (45.0%)	6 (29.2%)	
Bone loss – 24 months	Yes	8 (35.0%)	13 (58.3%)	0.143
	No	14 (65.0%)	9 (41.7%)	

The measured mean values of MBL (in mm) were categorized into bone loss (> 0 mm) and bone gain (< 0 mm) for each respective time point and are presented in Table 5. The values are divided into eight groups, ranging from the group with − 1 to -0.5 mm (bone gain) in increments of 0.5 mm, up to the group with > 2 to 2.5 mm (maximum bone loss). A value of ± 0 mm was assigned to the bone gain category for statistical analysis purposes. The most frequent cases of bone loss occurred within the groups > 0–0.5 mm and > 0.5–1.0 mm. In contrast, bone gain cases were limited to the groups ranging from − 1 to -0.5 mm and − 0.5 to 0 mm, with the majority falling within the − 0.5 to 0 mm range. In 10 patients, bone gain was detected consistently across all three observation periods, 8 of whom had received additional augmentation during the surgical procedure.

**Table 5:** Distribution of all measured mean MBL values categorized by groups over the observation periods

Marginal Bone Loss / Bone Gain (mm)	Values after 6 Months		Values after 12 Months		Values after 24 Months	
	*n*	%	*n*	%	*n*	%
-1 to -0.5	2	4.5	3	6.8	2	4.5
-0.5 to 0	12	27.3	12	27.3	19	43.2
0	1	2.3	1	2.3	2	4.5
> 0 to 0.5	19	43.2	19	43.2	11	25
> 0.5 to 1.0	6	13.6	5	11.3	6	13.6
> 1.0 to 1.5	1	2.3	3	6.8	2	4.5
> 1.5 to 2.0	3	6.8	0	0	1	2.3
> 2.0 to 2.5	0	0	1	2.3	1	2.3
**Total**	**44**	**100**	**44**	**100**	**44**	**100**

### Clinical evaluations

**Fig. 8:**
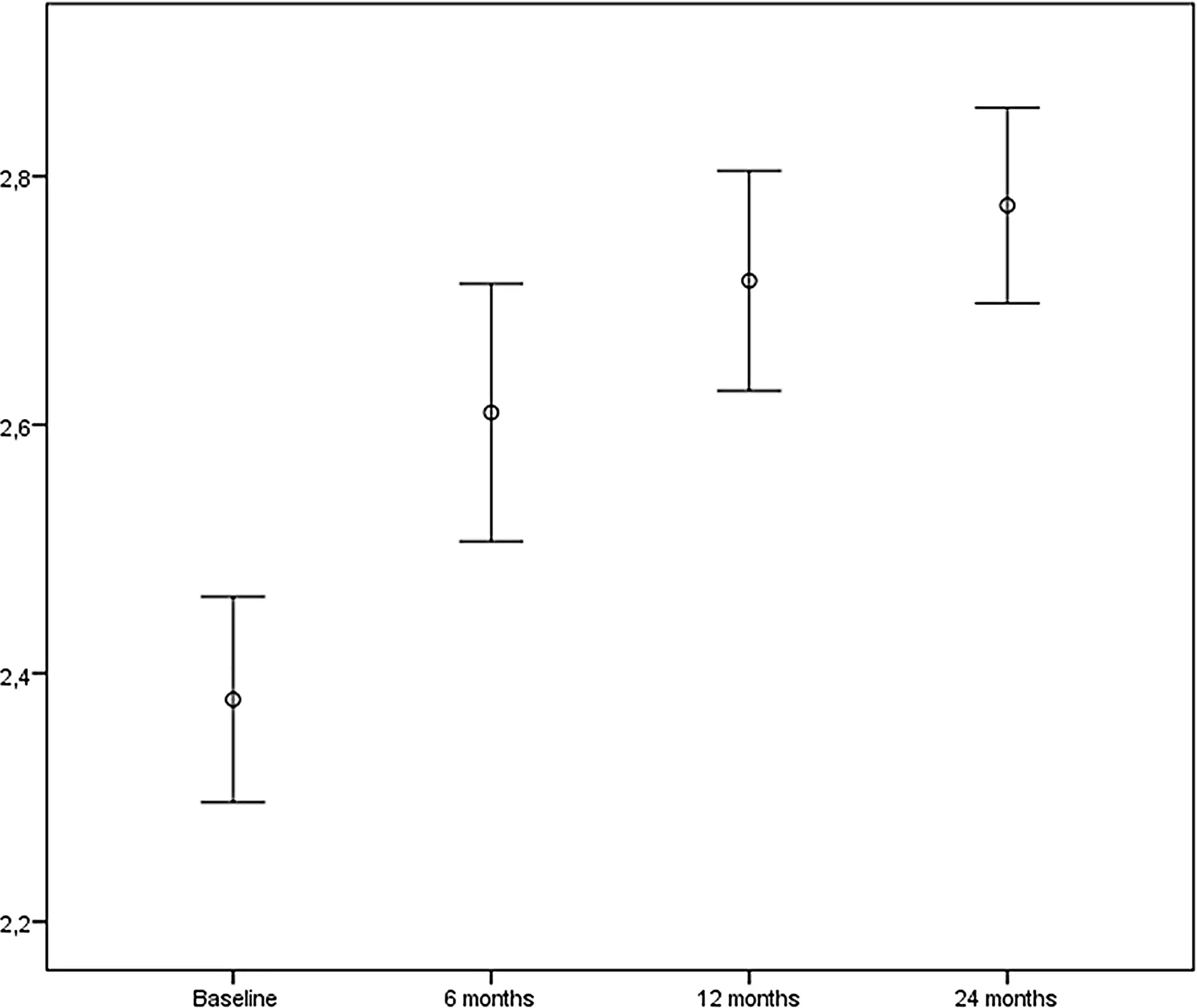
Mean probing depths at the time points baseline, 6, 12, and 24 months (95% CI)

**Fig. 9:**
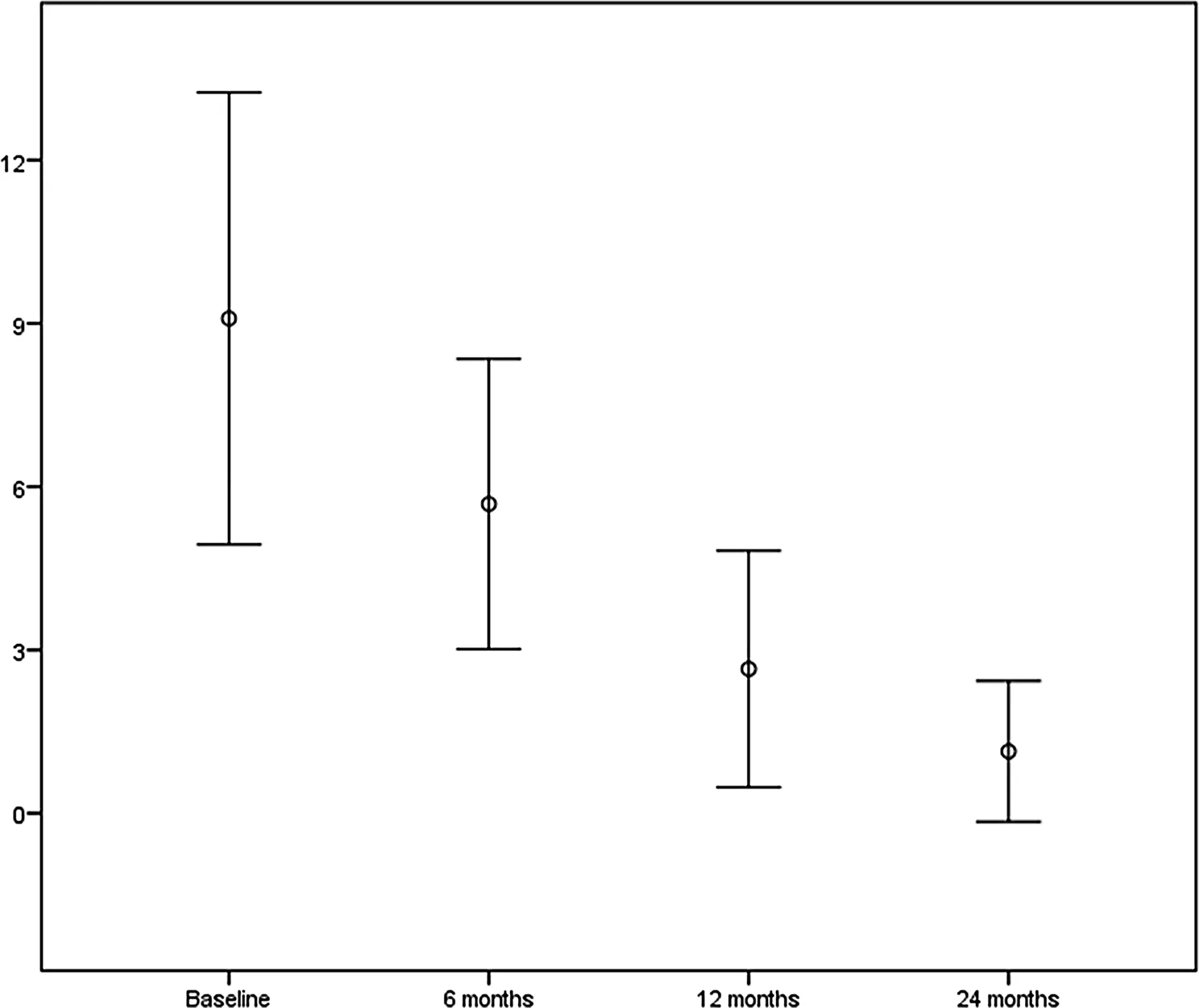
95% CI of the changes in mean BOP values at the follow-up time points

The clinical parameters, PD and BOP, are shown in Table 6 and Figs. 8 and 9. The mean PD was measured after 24 months at 2.78 mm. There was a significant change (*p* < 0.001) in the mean values compared to baseline at 6, 12, and 24 months, with effect sizes (Cohen’s d) of 0.74, 1.11, and 1.51 respectively, indicating moderate to large effects. Additionally, significant changes were found between 6 and 12 months (*p* = 0.042) and between 6 and 24 months (*p* < 0.001), while differences between 12 and 24 months were not statistically significant (*p* = 0.129). As BOP values were not normally distributed, significant differences were observed between baseline and 24 months (*p* = 0.002; Cohen’s d = 0.66), as well as between 6 and 24 months (*p* = 0.003; Cohen’s d = 0.54). Effect sizes for all comparisons are presented in Table 6. No biological complications were observed. Technical complications occurred in two implants. During the prosthetic restoration, a fracture of the abutment screw was noted, as well as an insufficient fit of the single crown on the abutment, which led to gap formation after the final prosthetic restoration. Applying the predefined success criteria (PD ≤ 4 mm, absence of BOP, no suppuration, MBL ≤ 1.7 mm), 41 of the 44 implants (93.2%) fulfilled all four criteria at the 24-month evaluation. Three implants did not meet the success threshold: two exceeded the MBL limit during the observation period (maximum 2.3 mm at 24 months, as shown in Table 5), and one demonstrated residual BOP at the 24-month visit without radiographic evidence of bone loss. None of these implants required removal. The implant survival rate therefore remained 100%, while the implant success rate was 93.2%.

**Table 6:** Mean values of clinical parameters PD and BOP at different time points, with effect sizes (Cohen’s d) versus baseline

Time Point	PD (mm) Mean ± SD	BOP (%) Mean ± SD	Cohen’s d (PD vs. baseline)	Cohen’s d (BOP vs. baseline)
Baseline	2.38 ± 0.27	9.1 ± 13.7	—	—
6 months	2.61 ± 0.34	5.7 ± 8.8	0.74	0.31
12 months	2.72 ± 0.29	2.7 ± 7.1	1.11	0.54
24 months	2.78 ± 0.26	1.1 ± 4.3	1.51	0.66

## Discussion

The aim of the present study was to investigate the survival and fracture rates, MBL, PD, and BOP of the peri-implant mucosa of a commercial, two-piece ATZ implant system. The 100% survival rate of the 63 implants (including SCs, FDPs and overdentures), as well as the absence of implant fractures, confirm the clinical reliability of the implant system examined within the observed period. Due to the heterogeneity of implant success criteria and classification systems reported in the literature, an overall implant success rate was deliberately not calculated; instead, the individual clinical and radiographic outcome parameters were reported separately.

The first reports on survival rates of two-piece ZrO₂ implants were published a decade ago by Becker et al. and Payer et al. [3, 45]. However, many of the systems investigated at that time are no longer market-available, limiting the transferability of these results to current implant systems. Therefore, studies involving currently available implants are of interest. Moreover, scientific data on two-piece ZrO₂ implants remain limited, underscoring the need for further research in this area [50]. Two studies on commercial, two-piece ZrO₂ implants reported survival rates of 96.5% [10] and 95.8% [3] after three and two years, respectively. In addition, a more recent study with 65 commercial, two-piece ceramic implants reported a survival rate of 98.5% after 12 months, highlighting the stability and reliability of certain implant systems [15]. Contrarily, Jennes et al. reported a survival rate of 60.9% after 12 months, with failures attributed to implant mobility at re-entry and after loading, as well as implant fractures [24]. These heterogeneous results highlight the need for further clinical studies on commercial two-piece systems.

A recent in vitro study on 32 implants highlights the fracture resistance of the two-piece ZrO_2_ implants of the same type used in our study. Both the implants and the carbon-fiber reinforced PEEK abutment screws showed no impairment in fracture resistance under various loading and hydrothermal aging protocols [32]. Therefore, the results of this clinical study are consistent with preclinical results reported in the literature. While peri-implantitis is considered the primary cause of titanium implant loss, its significance for ZrO_2_ implants is debated [17, 26, 33, 45]. In vitro and animal studies have been conducted on this topic, providing important insights [29, 49]. A prospective study investigating patients each receiving one ZrO_2_ and one titanium implant demonstrated more pronounced peri-implant soft tissue inflammation and higher bacterial colonization around titanium implants [14]. These findings were confirmed in a randomized clinical trial involving 42 patients, each with one ZrO_2_ and one titanium implant [7]. More studies are needed to further characterise the peri-implant immune response and the long-term risk of peri-implantitis around two-piece ZrO₂ implants in clinical settings.

Baseline marginal bone level was recorded at the time of implant exposure (second-stage surgery). Positive values indicate bone resorption, whereas negative values indicate bone apposition relative to this reference point. Remodelling during the submerged healing phase (approximately 4–6 months) is therefore not captured in the reported MBL values, and comparisons with studies using implant placement as baseline should be interpreted with caution. Furthermore, the absence of standardized radiographic holders may have affected measurement reproducibility and accuracy. The MBL values were increased within the first 6 months. Bone loss decreased at 12 and 24 months, with only a slight increase observed between 12 and 24 months. This temporal pattern of bone loss, with the greatest reduction occurring in the first 6–12 months, has been widely reported in the literature [2, 18, 22, 38, 39, 44]. Most studies on two-piece titanium implants agree that marginal bone loss should not exceed 1 mm during the first year following prosthetic restoration [12, 38, 46]. However, a unified recommendation for acceptable MBL values over time, especially for ZrO_2_ implants, is still missing.

The majority of MBL studies have focused on one-piece zirconia implants. A meta-analysis of 376 predominantly one-piece ceramic implants found a mean MBL of 0.7 mm (95% CI: 0.4–1.0 mm) after one year [49]. It should be noted that one-piece and two-piece zirconia implant designs differ in clinically relevant respects, including the presence of an implant–abutment connection with potential for micromotion and microgap formation, which may independently influence crestal bone behaviour. Direct numerical comparison of MBL values across these designs therefore carries inherent limitations and should be interpreted with caution. In the present study, bone loss after six months was minimal. No statistically significant differences in MBL were detected across observation periods (*p* ≥ 0.062), consistent with the Results section. Comparable results were reported in a prospective clinical study of 50 one-piece ceramic implants, where the greatest bone loss, of 0.71 mm (± 0.67 mm, *p* < 0.001), was also observed between implantation and prosthetic delivery [31].

In this study, bone loss was not significantly influenced by patients’ gender. Furthermore, it was greater in the maxilla than in the mandible, though the differences remained within the range of random variation. The authors attribute this tendency to the higher bone mineral density in the mandible, which provides greater resistance to mechanical load and leads to slower remodeling in the crestal region [38]. A cohort study involving 48 one-piece zirconia implants found no differences in MBL between the maxilla and mandible after five years [31]. Similarly, an investigation of 26 one-piece zirconia implants over a ten-year period reported no jaw-related differences in bone loss [9].

Notably, in the present study, mean MBL was consistently lower in cases with additional bone augmentation across all observation periods compared to cases without augmentation. Although these differences did not reach statistical significance, this consistent trend may indicate a potentially beneficial influence of augmented bone on peri-implant marginal bone stability. Interestingly, within the first six months, MBL was significantly higher—by 29%—in cases without bone augmentation compared to those with augmentation. This early difference may reflect a more favorable remodeling response in augmented sites; however, given the retrospective design and limited sample size, these findings should be interpreted with caution and do not allow for definitive causal conclusions. Similar recommendations have been described in the literature for titanium implants [11]. However, a meta-analysis of 376 commercial ZrO₂ implants found no influence of additional bone augmentation on marginal bone loss [49]. These results were confirmed by a systematic review of two randomized and seven prospective studies, which likewise showed no changes in MBL associated with bone augmentation procedures [47]. Further studies are needed to evaluate the effect of bone augmentation on MBL.

The mean PD remained within a clinically unremarkable range at all time points (2.38 ± 0.27 mm to 2.78 ± 0.26 mm). Although a continuous increase in mean PD values was observed over time, the change was minimal. These results are consistent with Roehling et al., which reported a mean PD of 3.0 mm after a five-year follow-up [50]. Furthermore, the BOP, demonstrated a marked decrease over the course of the follow-up examinations. After 24 months, all 44 implants showed no signs of gingival inflammation. In addition, none of the implants exhibited any indications of peri-implant mucositis or peri-implantitis. All implants met the criteria for healthy peri-implant tissues [5]. Within the scope of this investigation, the ZrO₂ implant system demonstrated high biocompatibility and stable integration of the peri-implant soft tissues.

The cement-free connection of the superstructure using VICARBO^®^ screws may account for the low technical complication rate observed in our study. This approach minimizes the risk of foreign bodies or cement remnants entering the sulcus during intraoral luting. The issue of cement-induced inflammation of the marginal mucosa around implants is repeatedly described as a complication in the literature [15, 34, 36]. Although a systematic review found no significant differences between screw-retained and cemented reconstructions in terms of survival and failure rates, technical and biological complications seem more frequent with cemented restorations [58].

Furthermore, a structured training process under the supervision of experienced operators prior to first-time use of novel implant systems is recommended. This can significantly reduce implant loss rates and complications attributable to a certain learning curve of the clinician [31]. Successful implant therapy requires more than correct implant placement and prosthetic restoration. Regular follow-up examinations at defined intervals are essential to prevent biological and technical complications and to ensure long-term survival rates. A formal a priori power calculation was not performed, as this was a retrospective analysis of an existing database. Post-hoc power estimation indicates that the sample was moderately powered for the primary MBL outcome. All findings should therefore be interpreted as exploratory; definitive conclusions regarding the superiority or equivalence of this implant system relative to comparators would require adequately powered prospective trials. Finally, the present study was financially supported by CeramTec GmbH, and one author (T.M.) served as a paid lecturer for the manufacturer’s distributor between 2014 and 2024. These relationships represent a potential source of performance and reporting bias inherent to industry-affiliated research. Several design features were implemented to mitigate this risk: patient data were fully anonymised before entry into the analytical database; radiographic measurements were performed by a calibrated examiner (T.M.) and independently verified by a second examiner (P.S.) with excellent inter-rater agreement (ICC = 0.95); and no selective outcome reporting was performed, as all pre-specified outcomes are reported. Readers should nonetheless interpret the findings in light of these funding relationships, while independent replication in investigator-initiated studies is warranted.

## Conclusions

The two-piece ZrO₂ implants supporting SCs examined in this study demonstrated stable and predictable outcomes with a high survival rate and no fractures. Both MBL and BOP values were similar to reference values reported in the literature for titanium implants. The low incidence of technical complications further underscores the reliability of this implant system for the investigated clinical indication.

## Data Availability

No datasets were generated or analysed during the current study.
